# Supplementary material for: Satellite quantification of enhanced methane oxidation applied to the stratospheric plume following Hunga Tonga-Hunga Ha’apai eruption
Source: Nat Commun. 2026 May 7;17:3746. doi: 10.1038/s41467-026-72191-4 (PMC13153224; doi:10.1038/s41467-026-72191-4)
Supplement: Supplementary file 1 — Supplementary Information [file 41467_2026_72191_MOESM1_ESM.pdf]

Supporting Information for

Satellite quantification of enhanced environmental oxidation of methane applied to the stratospheric plume following the Hunga Tonga-Hunga Ha'apai eruption

Maarten M.J.W. van Herpen*, Isabelle DeSmedt, Daphne Meidan, Alfonso Saiz-Lopez, Matthew S. Johnson, Thomas Röckmann, Jos de Laat

*Corresponding author. Maarten M.J.W. van Herpen.
Email: maarten@acacia-ii.com

This PDF file includes:

Supporting text
Figures S1 to S22
Tables S1 to S3
SI References

Other supporting materials for this manuscript include the following:

Datasets S1
Datasets S2

Supporting Information Text

Calculation of primary Cl production through HOCl reactive uptake

The peak rate of HOCl formation for Jan-21 is 2.2 × 10^6 $cm^{-3}s^{-1}$ based on peak MLS observations of ClO (0.7 ppb) and HO_2 (0.7 ppb). This HOCl formation rate is 10× higher than the 2 × 10^5 $cm^{-3}s^{-1}$ in the model output of Zhu et al. *(1)*, and the rate would be even 6x lower if we use the HO_2 concentration modelled by Zhu.

The fraction of HOCl production that leads to chlorine amplification depends on the competition between HOCl photolysis rate (2.3 × 10^{-4} s^{-1}) and reactive uptake rate (first order rate of 2.8 × 10^{-5} s^{-1} based on the maximum observed aerosol surface area density of 2.9 × 10^{-6} cm^2 cm^{-3}, a theoretically highest possible reaction probability γ=1.3 × 10^{-3} and thermal velocity v = 298 m/s, from Evan et al. (*2)*, due to which approximately 11% of HOCl production may lead to amplification. This means that the maximum Cl production through this mechanism is 2.5 × 10^5 $cm^{-3}s^{-1}$, while our observed value is similar at 3 × 10^5 $cm^{-3}s^{-1}$.

Considering that the reaction probability γ for HOCl + HCl is highly dependent on H_2O concentration and temperature (increasing 5 orders of magnitude from 5 ppm to 350 ppm H_2O, and 1 order of magnitude from 218 K to 222 K), it is possible that this reaction could be fast enough to explain some of the Cl production in the HTHH plume, but it cannot explain our observation that Cl production is sustained over multiple days, while H_2O concentrations are decreasing. Observation of HOCl could help, but unfortunately HOCl MLS observations are not suitable for scientific use at this pressure level.

We note that by Jan-25 the H_2O enhancement becomes partly separated from the HCHO/ClO/HO_2 enhancement (see Fig. S19). Also a vertical separation of the H_2O and aerosol layer was observed (3). This does not fit with an aerosol-based Cl source that depends on H_2O concentration, and is a strong argument why HOCl reactive uptake cannot explain the long-term primary chlorine production implied by our observed HCHO enhancement.

Calculation of primary Cl production through HOBr reactive uptake

For Jan-21 we observed a peak BrO enhancement of 2.5 × 10^{13} molec/cm^2, which corresponds with an estimated average concentration of 1.3 × 10^8 cm^{-3} (0.2 ppb). Combined with the peak MLS observations of HO_2 (0.7 ppb) the peak rate of HOBr formation for Jan-21 is 3.2×10^6 $cm^{-3}s^{-1}$.

The reactive uptake coefficient for HOBr is strongly dependent on temperature. Based on MLS observations the temperature of C1b_21 was 220 K *(4)* (see figure S11). At this temperature the maximum possible reaction probability γ = 1 × 10^{-3} based on Zhang et al. *(5)*.

The fraction of HOBr production that leads to chlorine amplification depends on the competition between HOBr photolysis rate (3.3 × 10^{-3} s^{-1}) and reactive uptake rate (first order rate of 1.6 × 10^{-5} s^{-1} based on the maximum observed aerosol surface area density of 2.9 × 10^{-6} cm^2 cm^{-3} (*2),* γ = 1 × 10^{-3} and thermal velocity v=219 m/s). This means approximately 0.5% of HOBr production may lead to amplification. This means that the maximum Cl production through this mechanism is 1.5 × 10^4 $cm^{-3}s^{-1}$, while our observed value is an order of magnitude larger at 3 × 10^5 $cm^{-3}s^{-1}$.

In addition to this, the observed correlation between HCHO and BrO was linear, while a non-linear correlation is expected if the mechanism depends on the combination of BrO, HO_2 and aerosols. For example, high BrO is associated with high aerosol optical depth and also associated with high HO_2, due to which the HCHO:BrO enhancement ratio would increase for higher BrO enhancements (which is not what we observed).

We therefore conclude that the observed correlation between HCHO and BrO enhancement is due to Br activation by Cl that is produced through another mechanism (instead of Br activating the Cl).

Figures

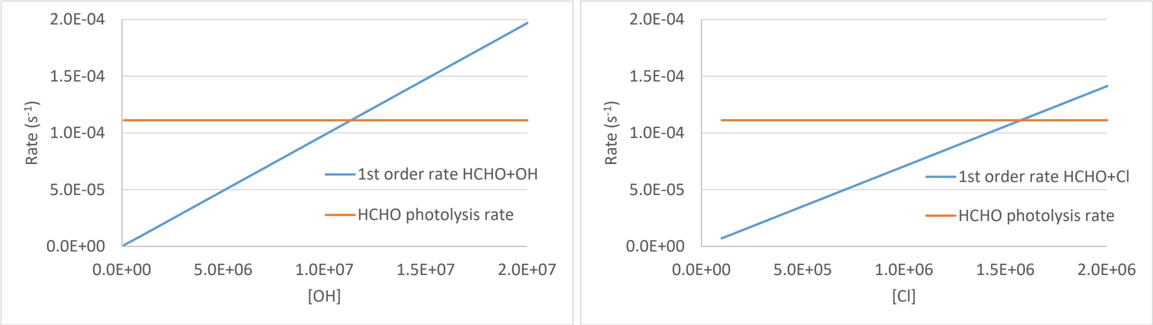

Fig. S1. Comparison of HCHO photolysis rate with the first order loss rate for varying [OH] and [Cl], showing that HCHO loss to OH and Cl is below 50% for a maximum concentration of 1×10^7 cm^{-3} for OH and 2×10^6 cm^{-3} for Cl.

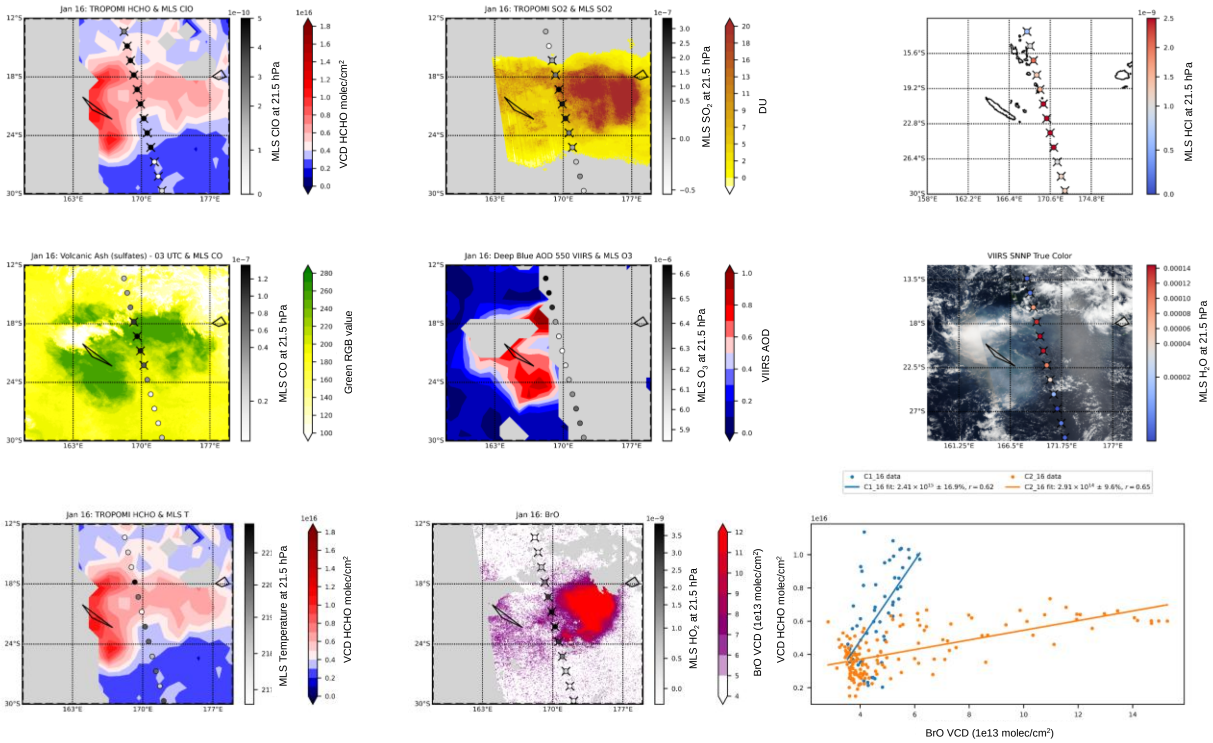

Fig. S2. Close-up view of C1_16 and C2_16. Top left: HCHO after median averaging to 1 degree resolution, compared with MLS ClO. Top middle: TROPOMI SO_2 compared with MLS SO_2. Top right: MLS HCl. Middle left: Modified EUMETSAT Geostationary Ring Volcanic Ash RGB – Multimission (downloaded on 1 May-2025), showing only the green channel to highlight the detection of the sulfate aerosol plume, compared with MLS CO measurements. Middle middle: VIIRS AOD compared with MLS O_3. Middle right: VIIRS True Color imagery from the Suomi NPP satellite (NASA/NOAA), accessed on 2025-6-4 via NASA EOSDIS Global Imagery Browse Services (GIBS) *(6)*, compared with MLS H_2O. MLS data marked with X did not pass quality screening. Bottom left: Repeat of top left, but now showing MLS Temperature instead. Bottom middle: TROPOMI BrO with overlayed MLS HO_2. Bottom right: correlation between BrO and HCHO.

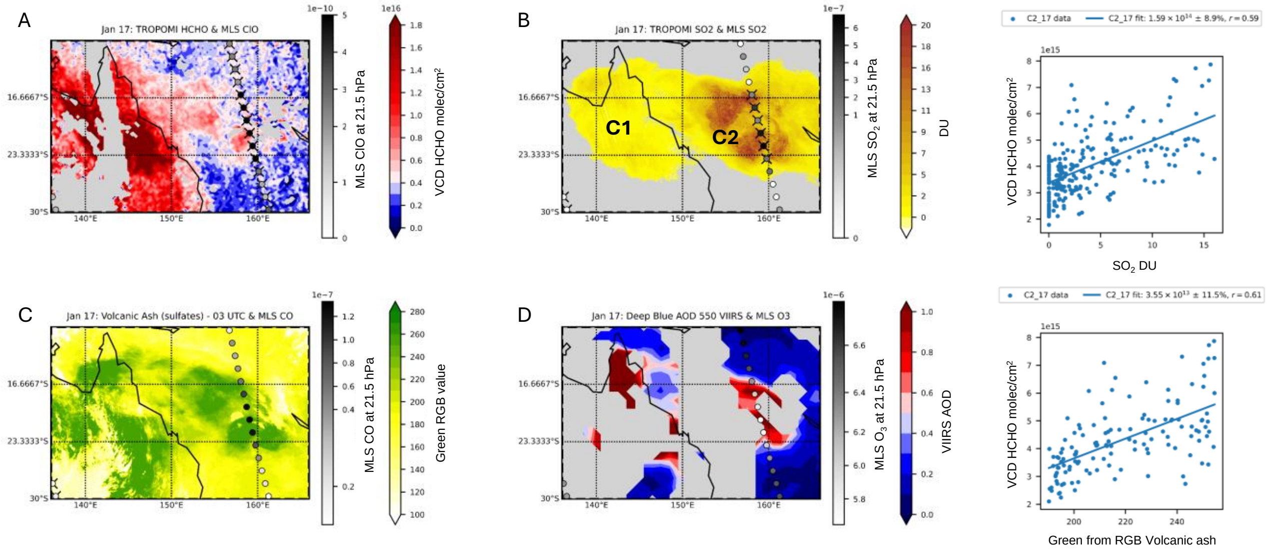

Fig. S3. Correlation between HCHO, SO_2 and aerosols within the HHTH plume on Jan-17. Only C2 could be confidently quantified, because C1 is located exactly above an Australian biomass burning source (which is a source of HCHO). A: HCHO VCD (without cloud correction) compared with MLS measurements of ClO. MLS data with an X did not pass quality screening. B: SO_2 column density compared with MLS SO_2 measurements. C: Modified EUMETSAT Geostationary Ring Volcanic Ash RGB – Multimission (downloaded on 1 May 2025), showing only the green channel to highlight the detection of the sulfate aerosol plume, compared with MLS CO measurements. D: VIIRS aerosol optical depth at 550 nm compared with MLS O_3 measurements. Right column: Correlation with HCHO.

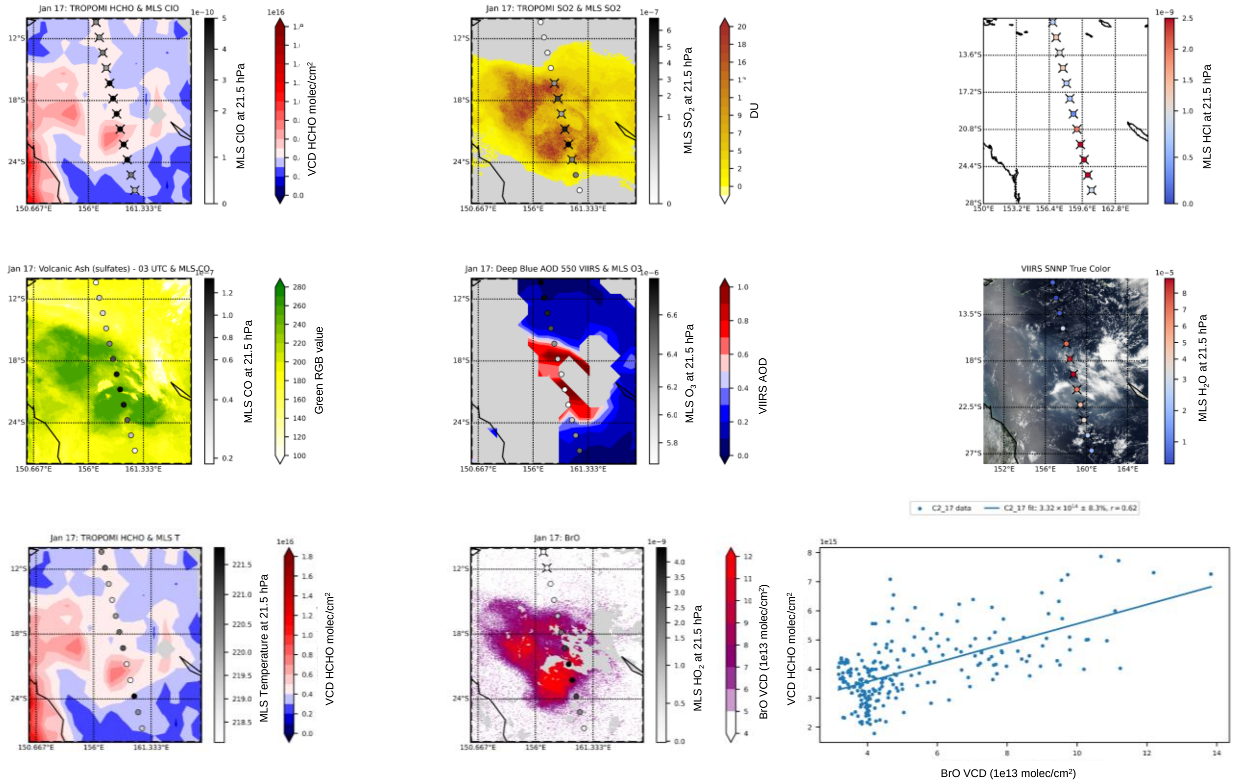

Fig. S4. Same as Figure S2, but for C2_17.

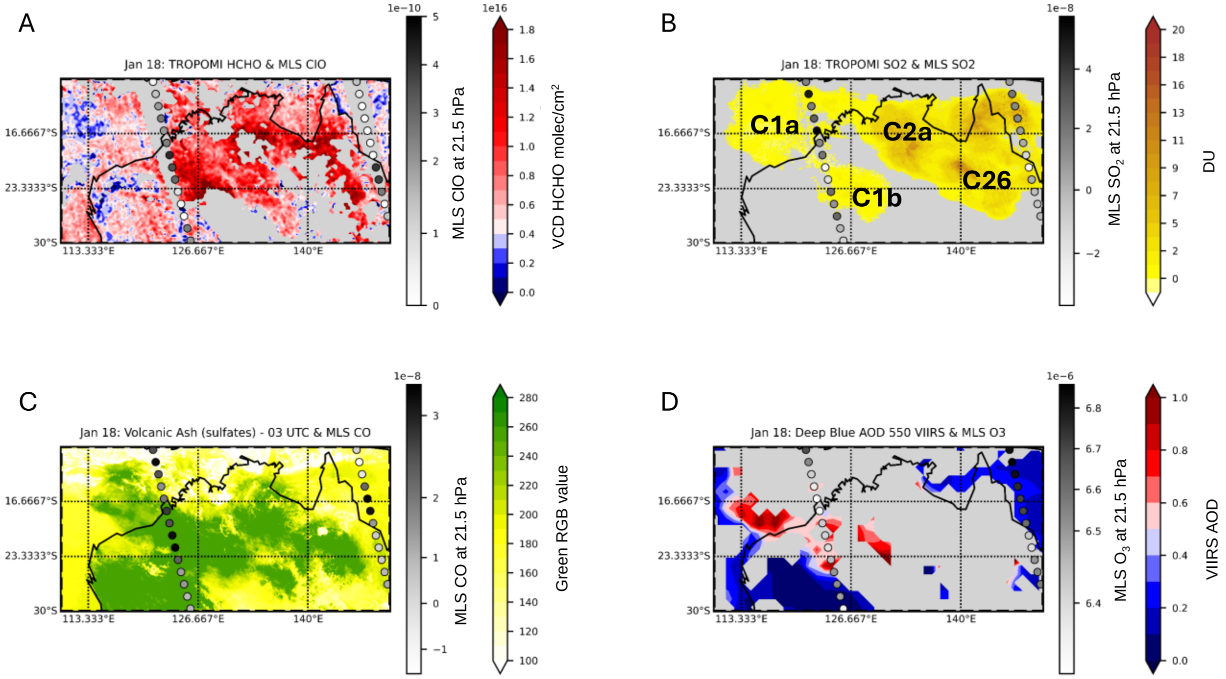

Fig. S5. Same as Figure S3, but for Jan-18. While the correlation appears to be clear between SO_2 and HCHO enhancement, we did not quantify for this day due to potential overlap with biomass burning regions.

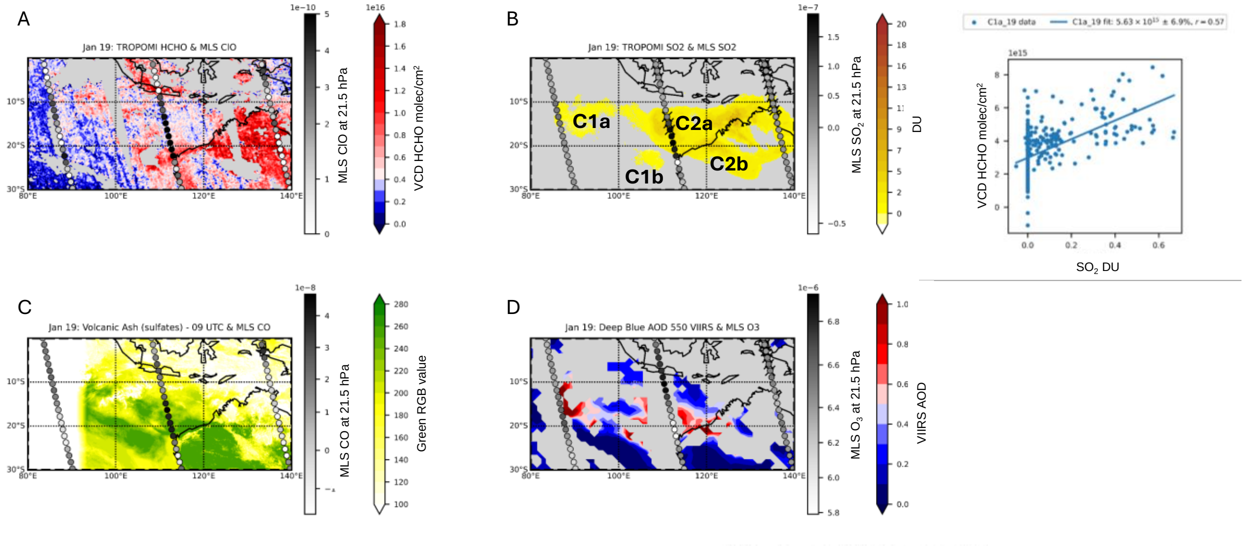

Fig. S6. Same as Figure S3, but for Jan-19. Only C1a could be confidently quantified, because the other clouds are potentially overlapping with continental HCHO sources. In addition, only SO_2 could be used to correlate C1a, because sulphate and AOD data are incomplete.

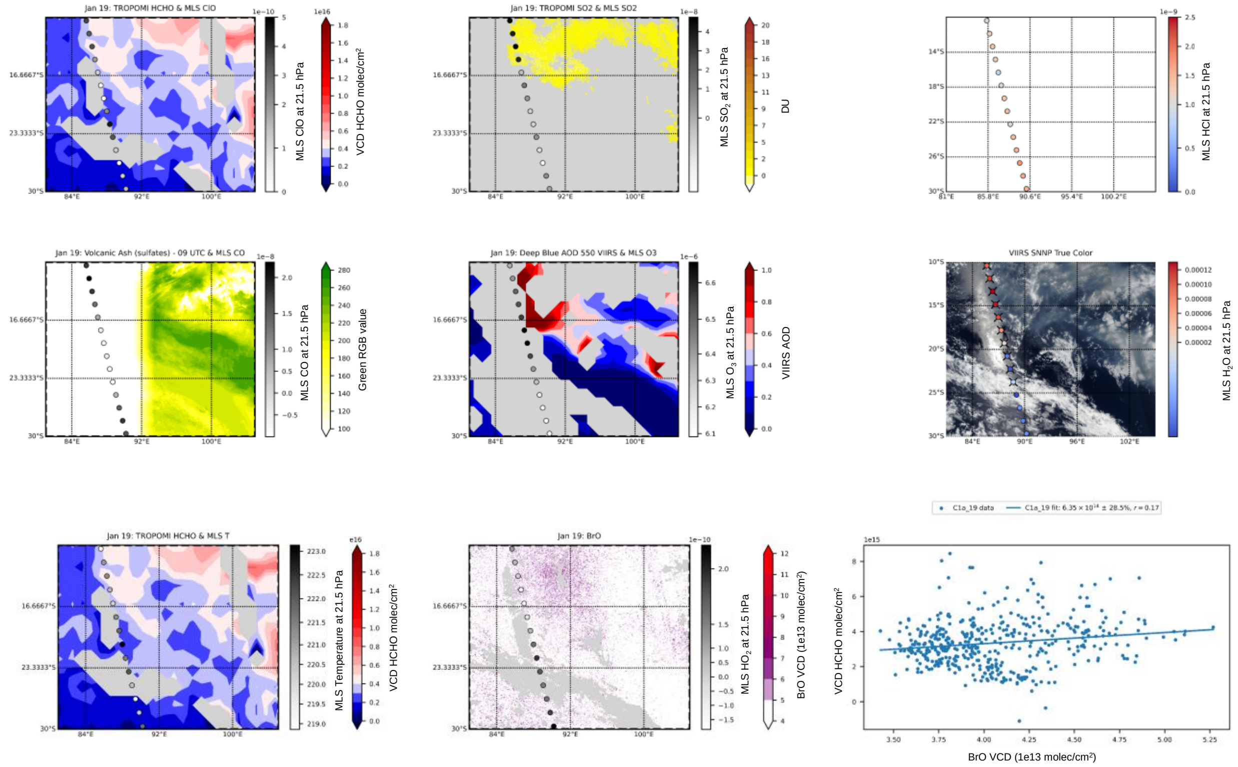

Fig. S7. Same as Figure S2, but for C1a_19.

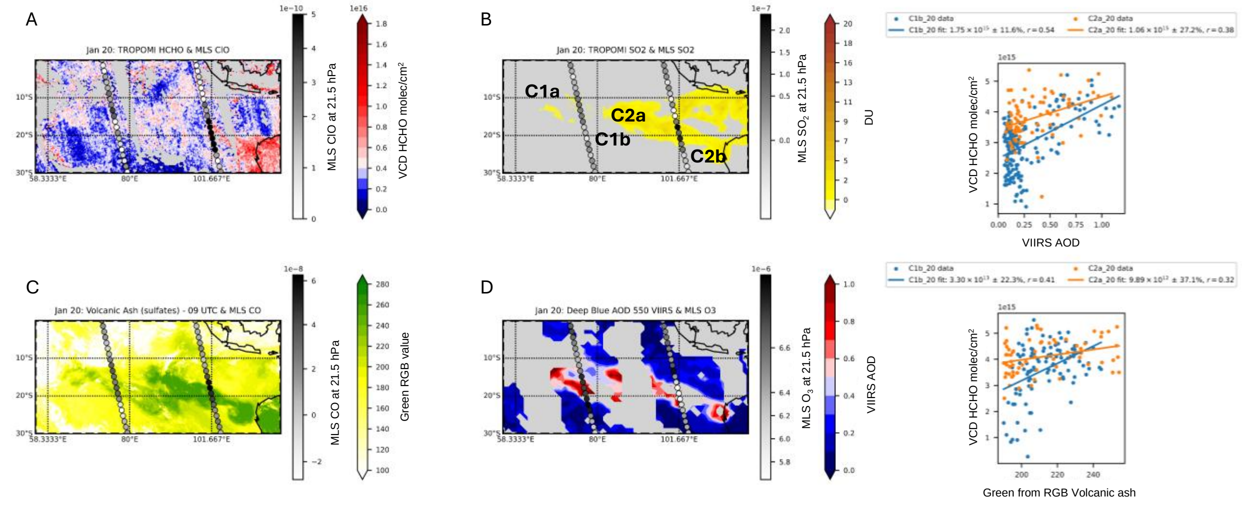

Fig. S8. Same as Figure S3, but for Jan-20. For C1 the SO_2 has mostly been removed. Only C1b could be confidently quantified, using AOD and sulphate. C1a could not be quantified due to clouds causing incomplete sulphate and AOD data. C2b could not be confidently quantified due to vicinity to continental biomass burning. Quantification of C2a was complicated due to overlap with C1b.

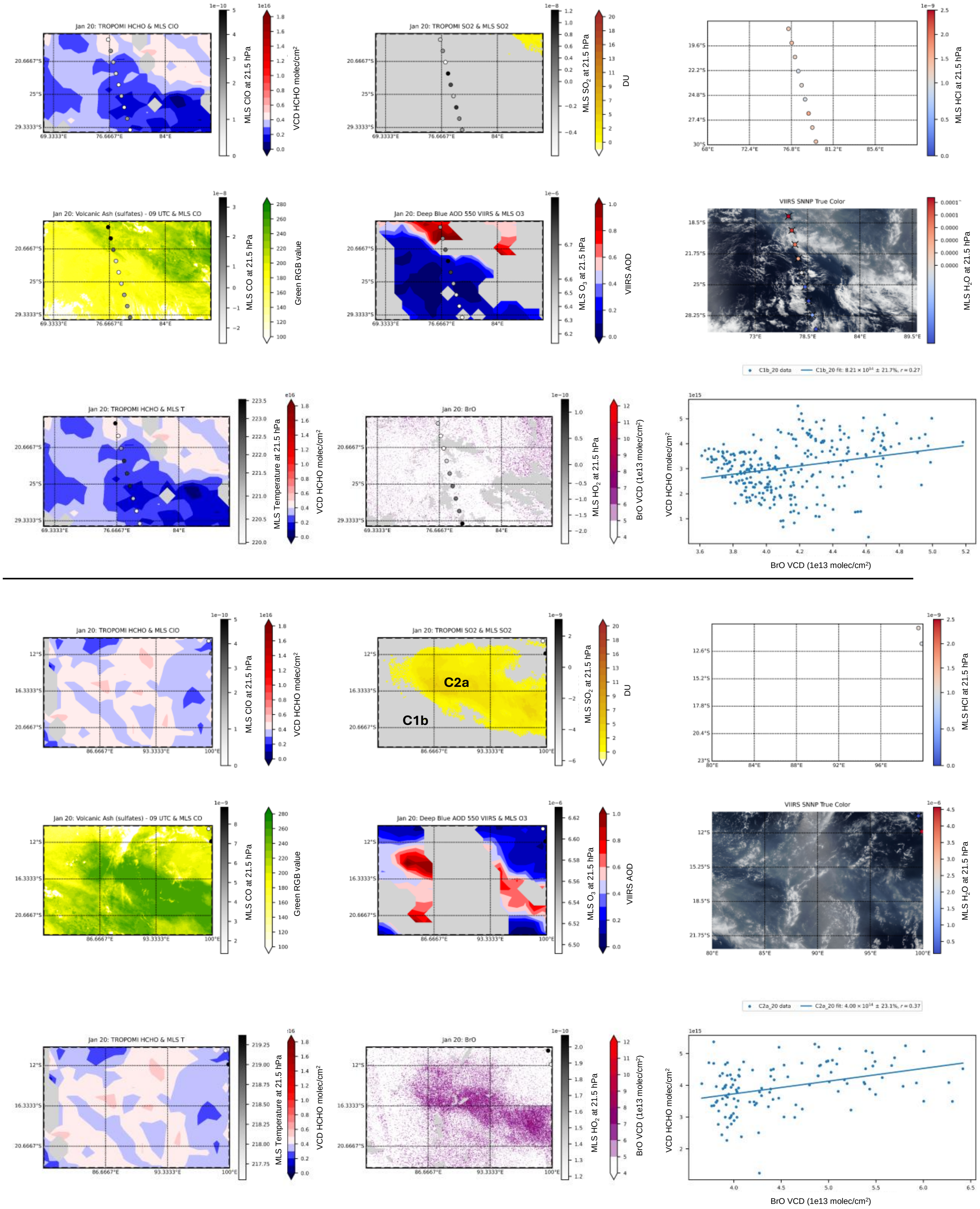

Fig. S9. Top 3 rows: same as Figure S2, but for C1b_20. Bottom 3 rows: same as Figure S2, but for C2a_20.

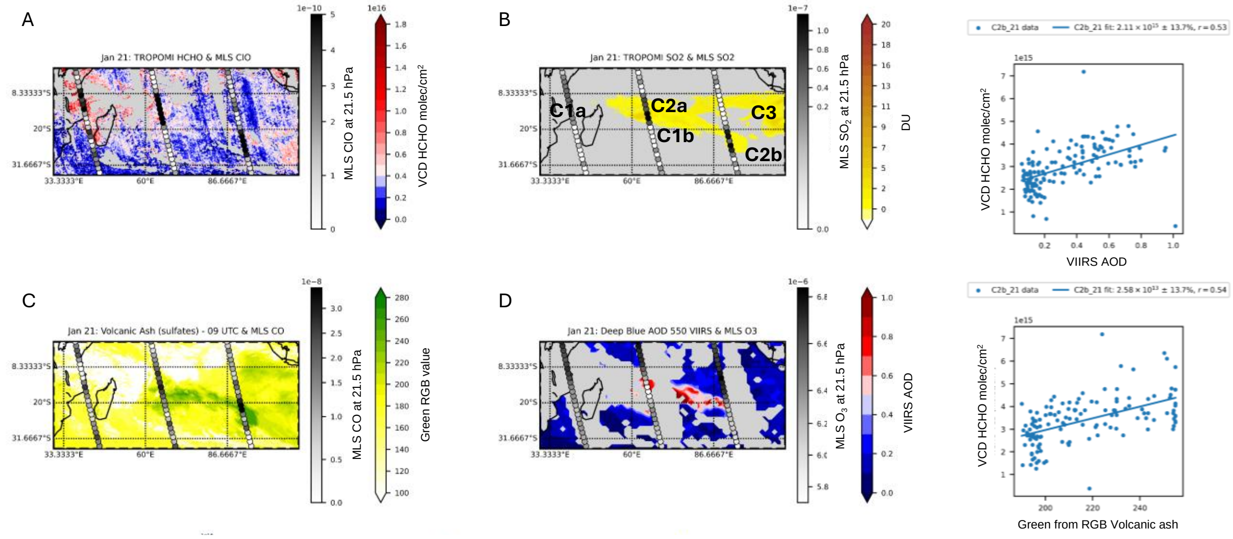

Fig. S10. Same as Figure S3, but for Jan-21. Only C2b could be confidently quantified, because of incomplete AOD and sulphate data for C1a, C1b and C2a, and overlap between C1b and C2a. For C3 we found no evidence of HCHO enhancement, but it is too close to Indonesia for a confident assessment.

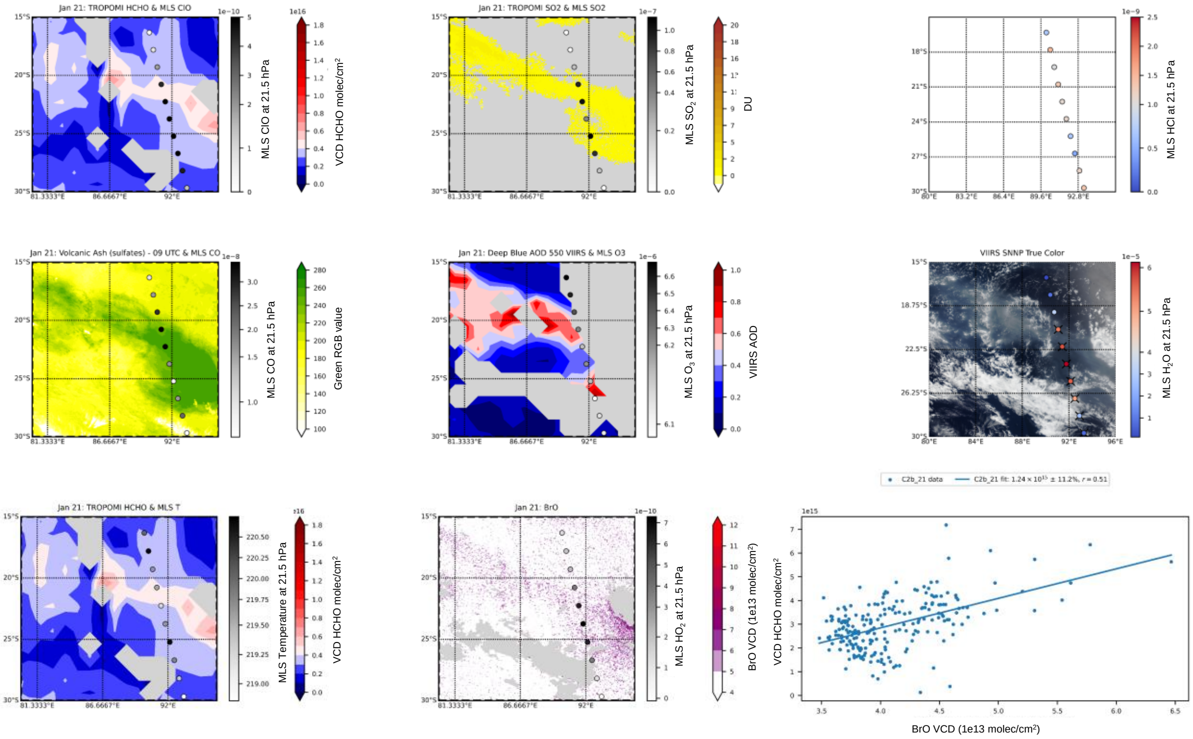

Fig. S11. Same as Figure S2, but for C2b_21.

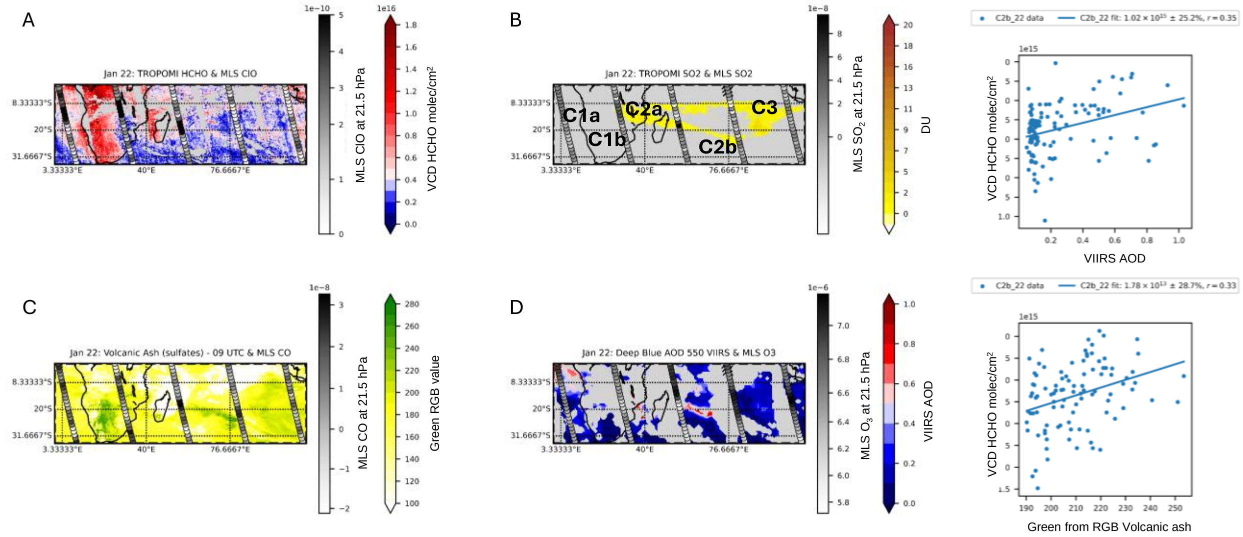

Fig. S12. Same as Figure S3, but for Jan-22. Only C2b could be confidently quantified, because of incomplete AOD and sulphate data and overlap with continental sources for the other clouds.

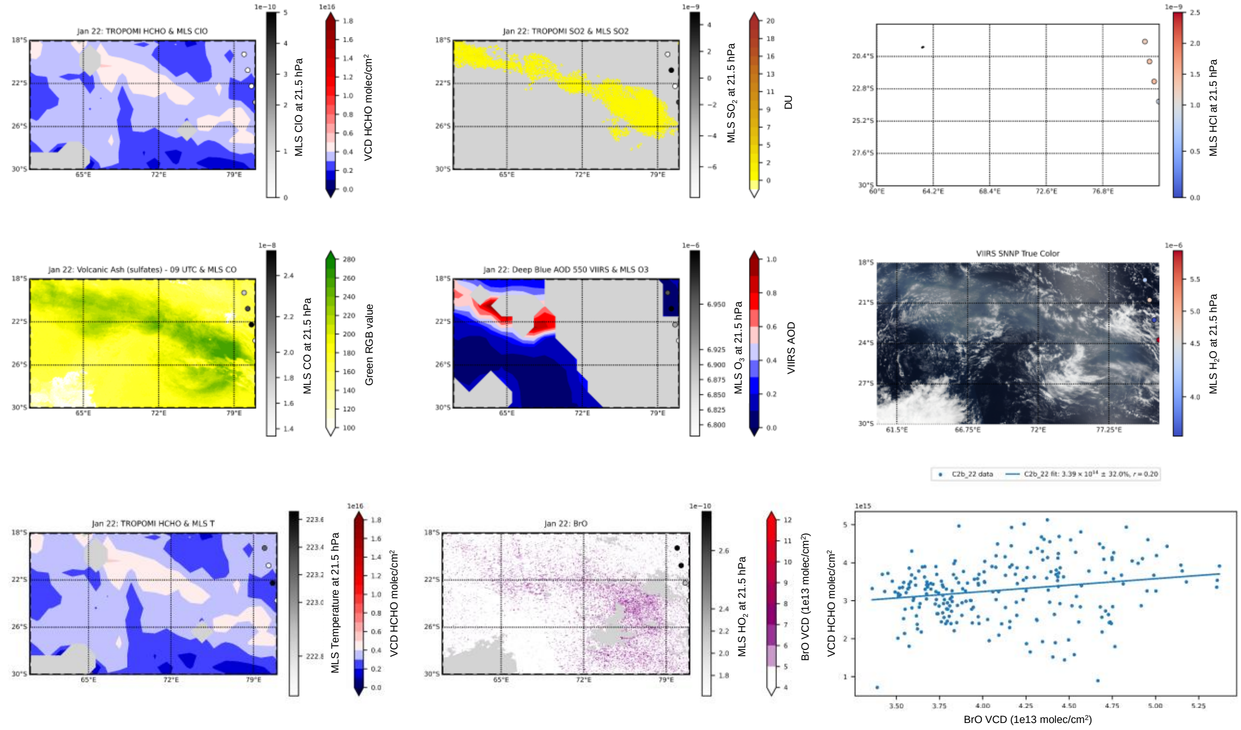

Fig. S13. Same as Figure S2, but for C2b_22.

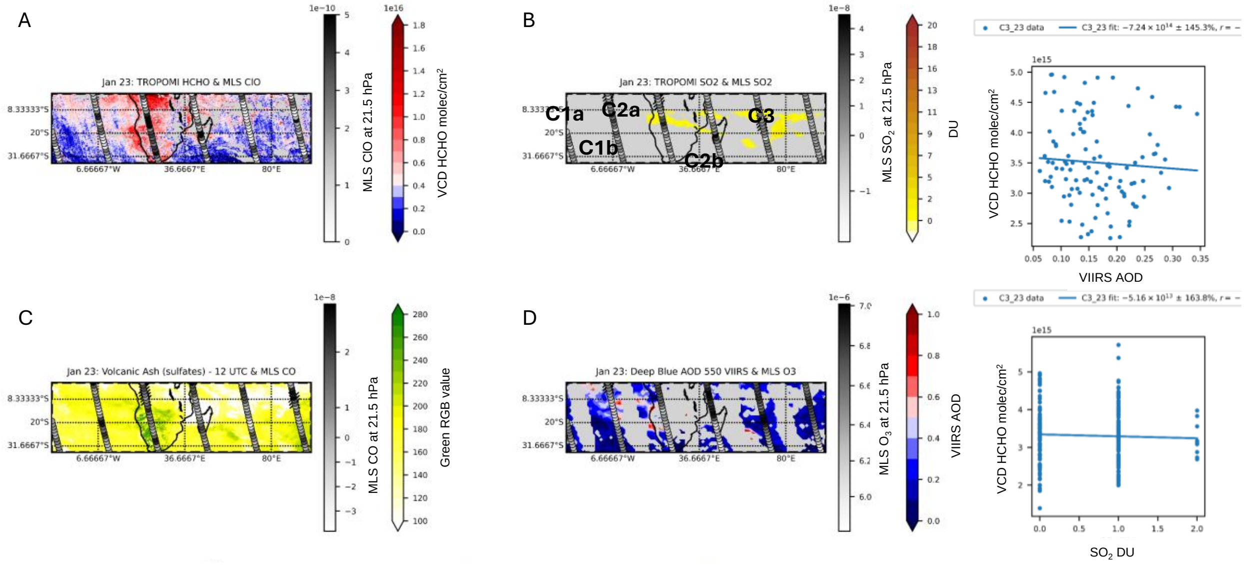

Fig. S14. Same as Figure S3, but for Jan-23. C1 and C2 could not be confidently assessed due to missing AOD data, clouds, and continental HCHO sources. C3 showed no HCHO enhancement, based on OAD and SO_2 correlation.

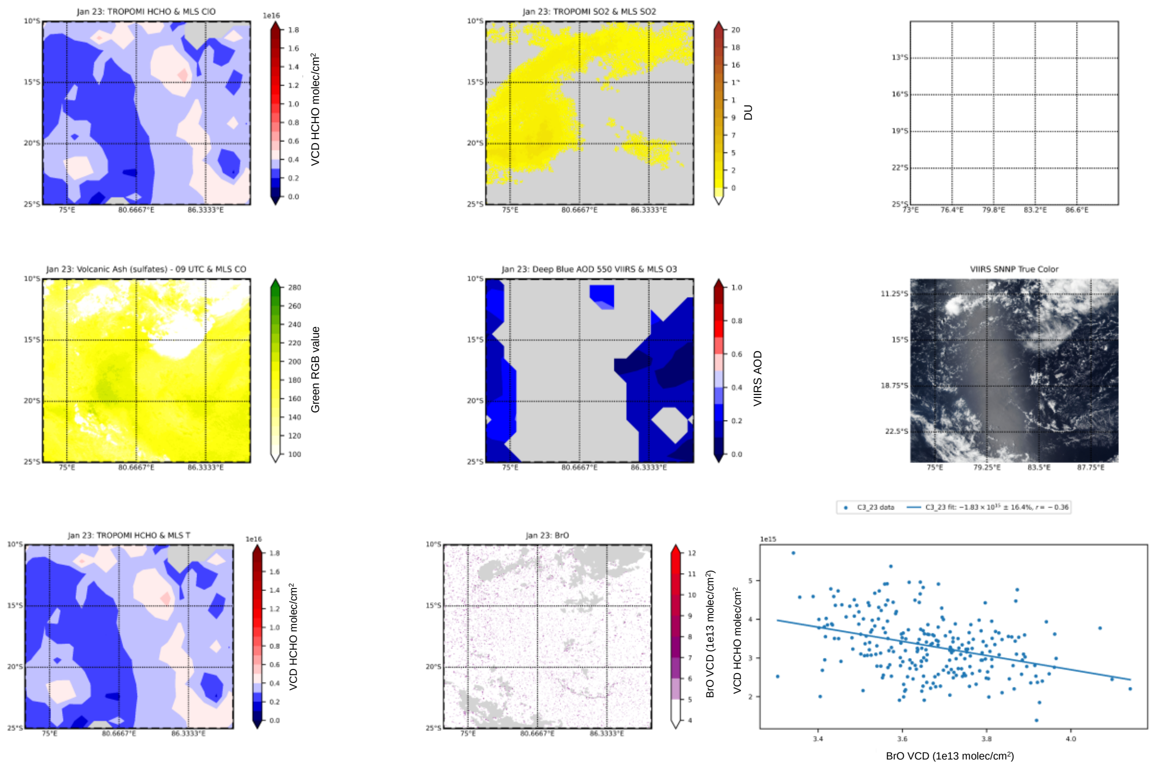

Fig. S15. Same as Figure S2, but for C3_23. This is an example of an SO_2 plume that has no AOD or BrO enhancement, and also has no HCHO enhancement.

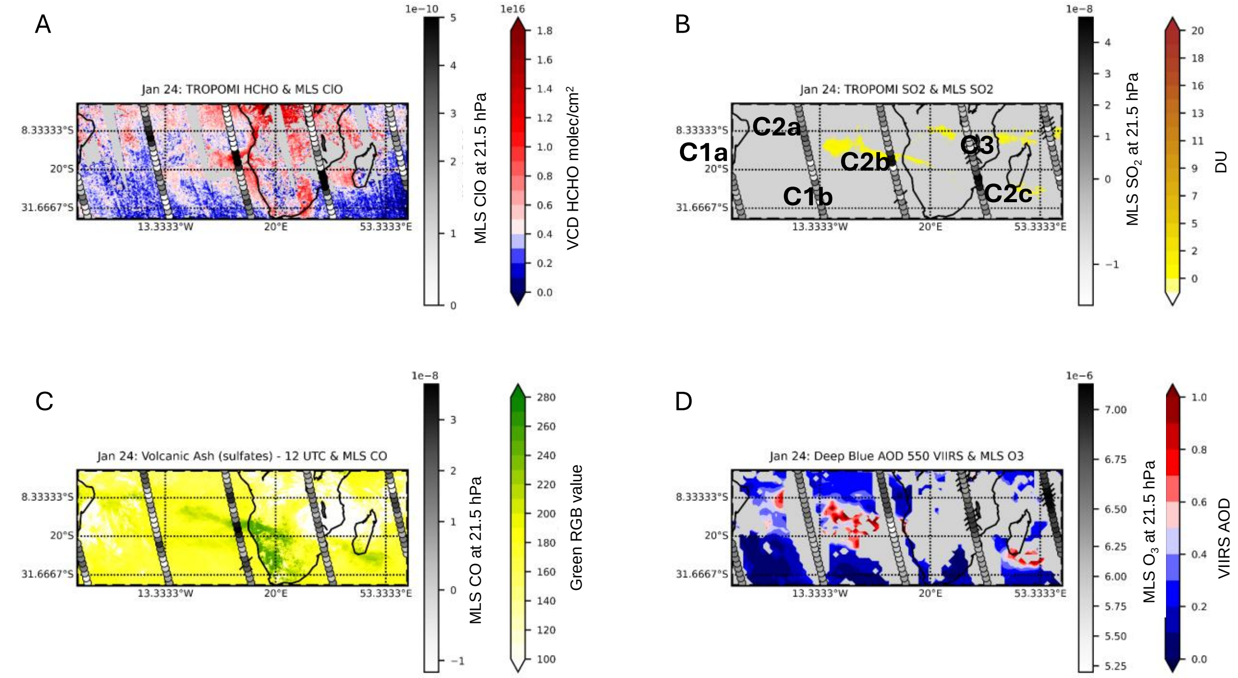

Fig. S16. Same as Figure S3, but for Jan-24. No HCHO enhancements could be confidently assessed. C1a is located over a land-based HCHO source. C2a shows enhancement but is also in the outflow of African biomass burning. C2b enhancement overlapping with continental outflow. C2c enhancement hard to quantify due to local sources. C3 now close too land to confidently assess.

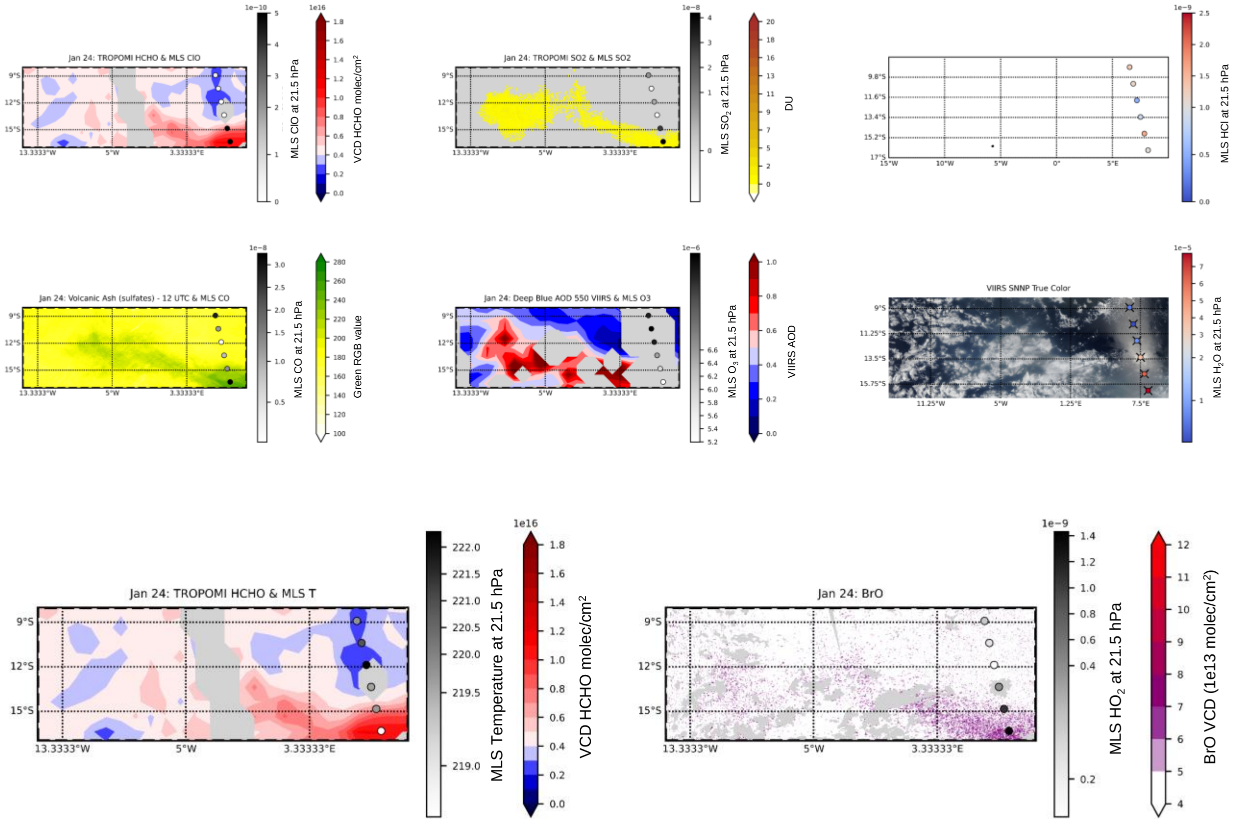

Fig. S17. Same as Figure S2, but for C2b_24. The correlation between HCHO/SO_2/sulfate/AOD appears to be there, but we could not confidently assess due to continental HCHO at the same location.

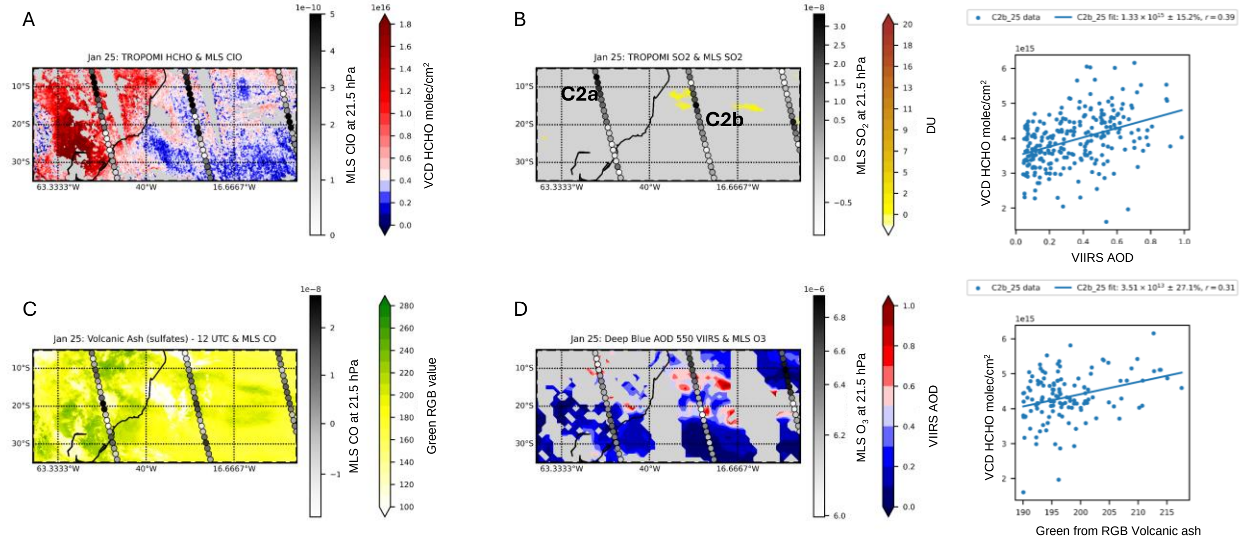

Fig. S18. Same as Figure S3, but for Jan-25. C2b was located over the Atlantic, and could partly be separated from the influence of continental outflow, showing correlation with AOD and sulphate.

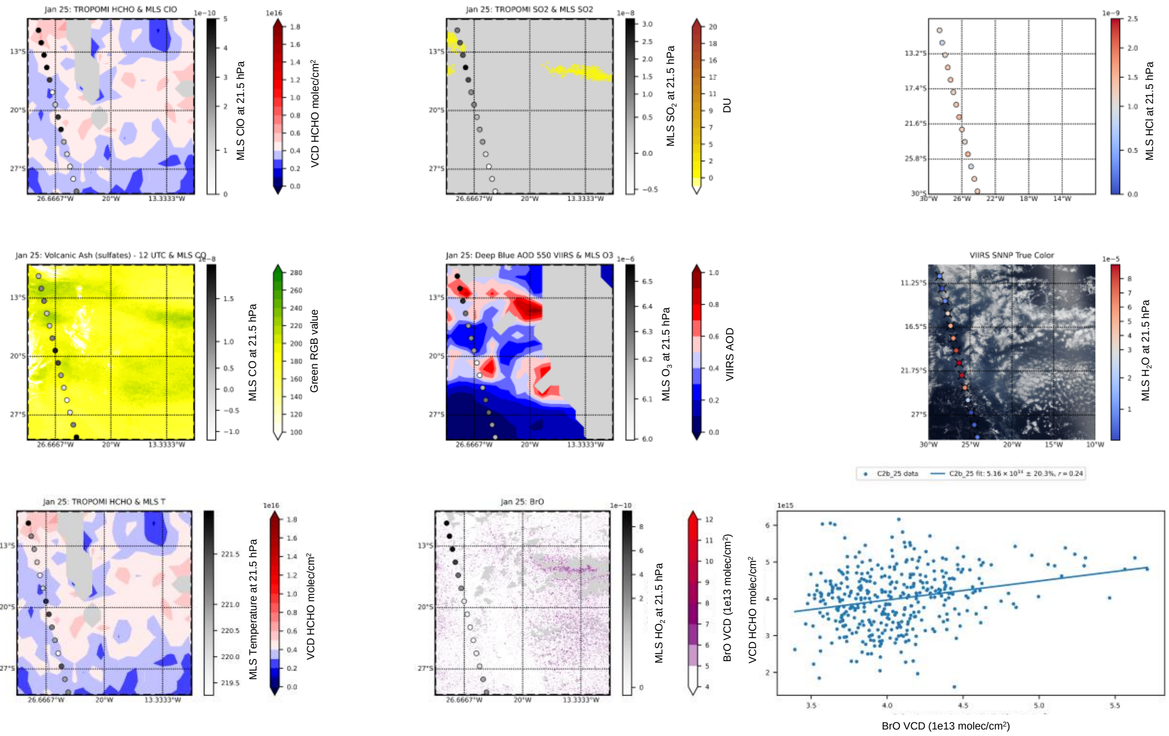

Fig. S19. Same as Figure S2, but for C2b_25. Note that the H_2O and the ClO measurements by MLS are no longer aligned, but displaced.

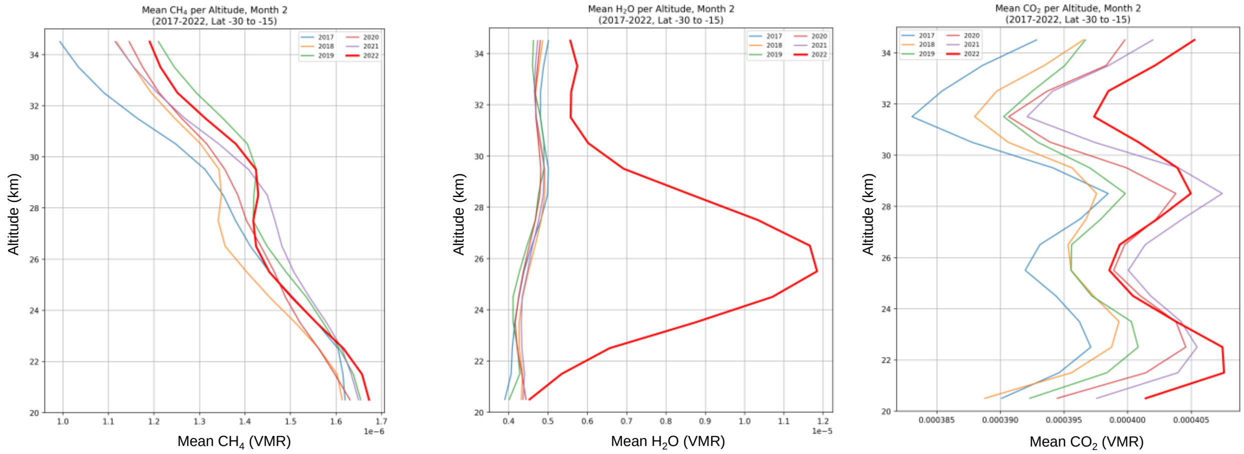

Fig. S20. Monthly average ACE-FTS observations between -30 and -15 latitude in Feb for several years. Left: CH_4 fluctuations of 0.05 ppm are very common, for example comparing 2017 and 2018 it can be seen that 2017 is 0.1 ppm below 2018 at 34 km altitude, while at 23 km altitude it is 0.05 ppm above 2018. Middle: H_2O showing an enhancement of 8 ppm in 2022. Right: No enhancement visible in CO_2 observations.

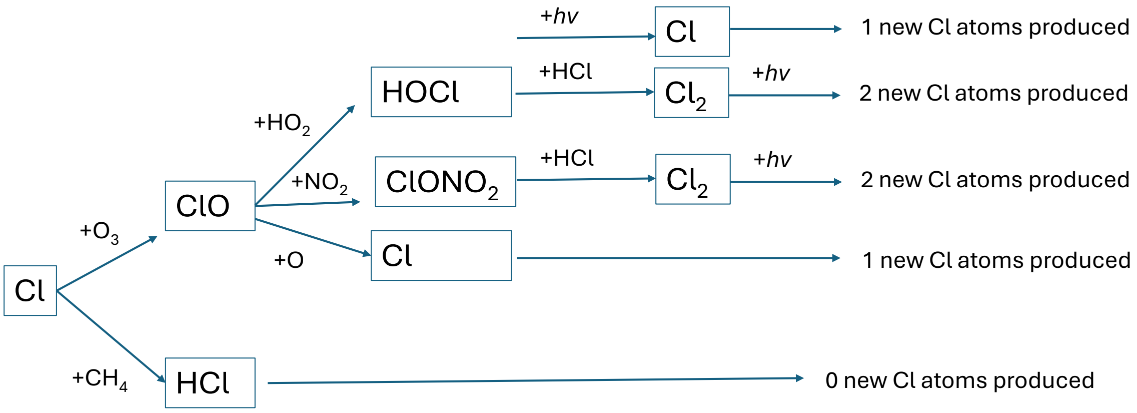

Fig. S21. Scheme of different mechanisms for chlorine activation.

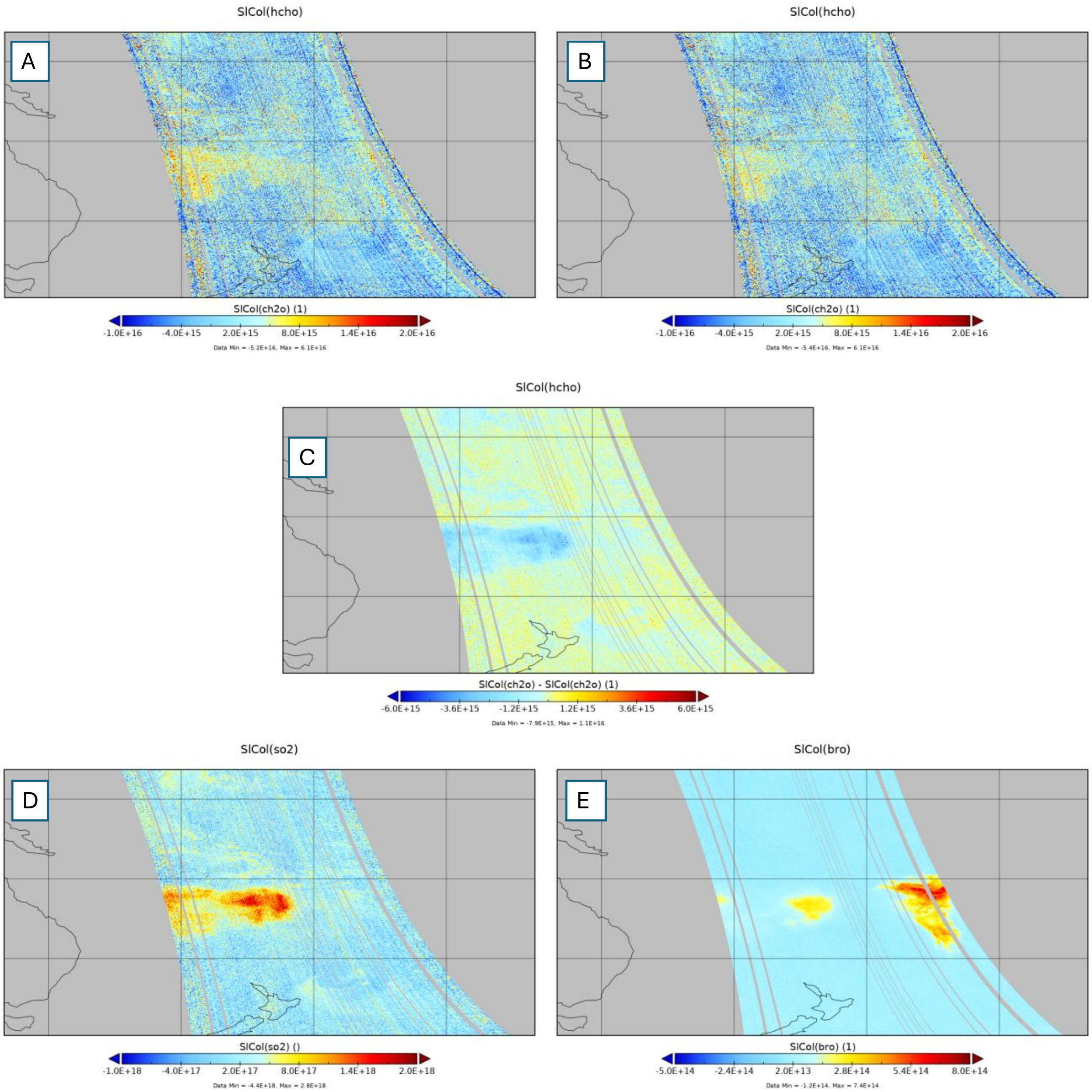

Fig. S22. A: Original HCHO SCD fit for Jan-16. B: SCD fit with SO_2 included. C: the difference between A and B shows that including SO_2 in the fits reduces the HCHO SCD mainly in C2, by about $2x10^{15}$ molec/cm^2. The difference is about $1x10^{15}$ molec/cm^2 in C1. D: Slant column density of SO_2. E: Slant column density of BrO.

Tables

Table S1. Overview of reactions relevant for HCHO.

#	Reaction	k at 298K and 1 atm (cm^3/s)	k at 220 K and 20 mbar (cm^3/s)	Reference
1	$CH_4 + OH \rightarrow CH_3 + H_2O$	$6.26\ 10^{-15}$	$9.01\ 10^{-16}$	*(11)*
2	$CH_4 + Cl \rightarrow CH_3 + HCl$	$1.03\ 10^{-13}$	$2.67\ 10^{-14}$	*(10)*
3	$HCHO + OH \rightarrow H_2O + HCO$	$9.38\ 10^{-12}$	$9.84\ 10^{-12}$	*(10)*
4	$HCHO + Cl \rightarrow HCl + CO + HO_2$	$7.32\ 10^{-11}$	$7.05\ 10^{-11}$	*(9)*
5	$O_3 + Cl \rightarrow ClO + O_2$	$1.22\ 10^{-11}$	$9.22\ 10^{-12}$	*(8)*
6	$ClO + HO_2 \rightarrow HOCl + O_2$	$6.88\ 10^{-12}$	$1.08\ 10^{-11}$	*(8)*<br>*(9)*
7	$BrO + HO_2 \rightarrow HOBr + O_2$	$3.32\ 10^{-11}$	$5.72\ 10^{-11}$	*(7)*

Table S2. Overview of photolysis reactions for HCHO.

#	Reaction
8	$HCHO + hv \rightarrow H + HCO$
9	$HCHO + hv \rightarrow H_2 + CO$

Table S3. Overview of cloud locations and corrections.

Observation	Coordinates	Cloud correction	AMF correction
C1_16	30 to 14 ^{0}S, 158 to 168 ^{0}E	1.2	4.85
C1a_19	30 to 10 ^{0}S, 81 to 105 ^{0}E	1.1	4.85
C1b_20	30 to 17 ^{0}S, 68 to 90 ^{0}E	0.9	4.85
C2_16	28 to 12 ^{0}S, 169 to 179 ^{0}E	1.1	4.85
C2_17	28 to 10 ^{0}S, 150 to 166 ^{0}E	1.1	4.85
C2a_20	10 to 10 ^{0}S, 80 to 100 ^{0}E	1.3	4.85
C2b_21	30 to 15 ^{0}S, 80 to 96 ^{0}E	1.2	4.85
C2b_22	30 to 18 ^{0}S, 60 to 80 ^{0}E	1.1	4.85
C2b_25	30 to 9 ^{0}S, 30 to 10 ^{0}W	1.1	4.85
C3_23	25 to 10 ^{0}S, 73 to 90 ^{0}E	0.8	4.85

Supplementary References

1. Zhu, Y., Portmann, R. W., Kinnison, D., Toon, O. B., Millán, L., Zhang, J., Vömel, H., Tilmes, S., Bardeen, C. G., Wang, X., Evan, S., Randel, W. J., and Rosenlof, K. H.: Stratospheric ozone depletion inside the volcanic plume shortly after the 2022 Hunga Tonga eruption, Atmos. Chem. Phys., 23, 13355–13367, https://doi.org/10.5194/acp-23-13355-2023, 2023.
2. Evan S, Brioude J, Rosenlof KH, Gao RS, Portmann RW, Zhu Y, Volkamer R, Lee CF, Metzger JM, Lamy K, Walter P, Alvarez SL, Flynn JH, Asher E, Todt M, Davis SM,

Thornberry T, Vömel H, Wienhold FG, Stauffer RM, Millán L, Santee ML, Froidevaux L, Read WG. Rapid ozone depletion after humidification of the stratosphere by the Hunga Tonga Eruption. Science. 2023 Oct 20;382(6668):eadg2551. doi: 10.1126/science.adg2551. Epub 2023 Oct 20. PMID: 37856589.

3. Schoeberl, M. R., Wang, Y., Ueyama, R., Taha, G., Jensen, E., & Yu, W. (2022). Analysis and impact of the Hunga Tonga-Hunga Ha'apai stratospheric water vapor plume. Geophysical Research lLetters, 49, e2022GL100248. https://doi.org/10.1029/2022GL100248
4. Millán, L., Santee, M. L., Lambert, A.,Livesey, N. J., Werner, F., Schwartz,M. J., et al. (2022). The Hunga Tonga-Hunga Ha'apai Hydration of the Stratosphere. Geophysical Research Letters, 49, e2022GL099381. https://doi.org/10.1029/2022GL099381
5. Zhang, J., Wang, P., Kinnison, D., Solomon, S., Guan, J., Stone, K., & Zhu, Y. (2024). Stratospheric chlorine processing after the unprecedented Hunga Tonga eruption. Geophysical Research Letters, 51, e2024GL108649. https://doi.org/10.1029/2024GL108649
6. NASA EOSDIS Global Imagery Browse Services (GIBS). VIIRS_SNPP_CorrectedReflectance_TrueColor (Suomi NPP VIIRS). Accessed via GIBS WMS (EPSG:4326), accessed 4 June 2025.
7. Atkinson, R., Baulch, D. L., Cox, R. A., Hampson Jr., R. F., Kerr, J. A., and Troe, J.: Evaluated kinetic and photochemical data for atmospheric chemistry: Supplement IV, IUPAC Subcommittee on Gas Kinetic Data Evaluation for Atmospheric Chemistry, J. Phys. Chem. Ref. Data, 21, 1125–1568, 1992.
8. Atkinson, R., Baulch, D. L., Cox, R. A., Crowley, J. N., Hampson, R. F., Hynes, R. G., Jenkin, M. E., Rossi, M. J., and Troe, J.: Evaluated kinetic and photochemical data for atmospheric chemistry: Volume III – gas phase reactions of inorganic halogens, Atmos. Chem. Phys., 7, 981–1191, https://doi.org/10.5194/acp-7-981-2007, 2007.
9. Atkinson, R., Baulch, D. L., Cox, R. A., Hampson Jr., R. F., Kerr, J. A., Rossi, M. J., and Troe, J.: Evaluated kinetic, photochemical, and heterogeneous data for atmospheric chemistry: Supplement V, IUPAC Subcommittee on Gas Kinetic Data Evaluation for Atmospheric Chemistry, J. Phys. Chem. Ref. Data, 26, 521–1011, 1997.
10. R. Atkinson, D. L. Baulch, R. A. Cox, J. N. Crowley, R. F. Hampson, Jr., J. A. Kerr, M. J. Rossi, and J. Troe, Summary of Evaluated Kinetic and Photochemical Data for Atmospheric Chemistry: Web Version (IUPAC Subcommittee on Gas Kinetic Data Evaluation for Atmospheric Chemistry, December 2001), pp. 1–56.
11. DeMore WB, Sander SP, Golden DM, Hampson RF, Kurylo MJ, Howard CJ, Ravishankara AR, Kolb CE, Molina MJ. Chemical kinetics and photochemical data for use in stratospheric modeling. Evaluation number 12. JPL Publication 97-4. Pasadena (CA): Jet Propulsion Laboratory, California Institute of Technology; 1997. p. 1-266.
