# Supplementary material for: Satellite quantification of enhanced methane oxidation applied to the stratospheric plume following Hunga Tonga-Hunga Ha’apai eruption
Source: Nat Commun. 2026 May 7;17:3746. doi: 10.1038/s41467-026-72191-4 (PMC13153224; doi:10.1038/s41467-026-72191-4)
Supplement: Supplementary file 2 — Description of Additional Supplementary Files [file 41467_2026_72191_MOESM2_ESM.pdf]

Description of Additional Supplementary Files:

Supplementary Data 1: contains all the observational data that was used to generate the correlations and totals shown in Table 1a and Table 1b of the manuscript. The file contains data for: lat, lon,HCHO VCD with cloud correction, SO2, HCHO VCD clear (without cloud correction), green value from of the EUMETSAT Volcanic 26 Ash RGB (representing sulfate aerosol), VIIRS AOD, TROPOMI AOD, modelled HCHO VCD from CAMS Reanalysis (model data), TROPOMI BrO.

Supplementary Data 2: contains MLS data used in the analysis.
